# Discovering Novel Small Molecule Compound for Prevention of Monoclonal Antibody Self-Association †

**DOI:** 10.3390/antib11020040

**Published:** 2022-06-08

**Authors:** Lok Hin Lui, Christopher F. van der Walle, Steve Brocchini, Ajoy Velayudhan

**Affiliations:** 1 UCL School of Pharmacy, University College London, London WC1N 1AX, UK; lok.lui.14@ucl.ac.uk (L.H.L.); s.brocchini@ucl.ac.uk (S.B.); 2 Dosage Form Design and Development, R&D BioPharmaceutical Development, AstraZeneca, Aaron Klug Building, Granta Park, Cambridge CB21 6GH, UK; wallec@medimmune.com; 3 Department of Biochemical Engineering, University College London, London WC1E 6BT, UK

**Keywords:** aggregation, antibodies, excipient, molecular dynamics, virtual screening, formulation design

## Abstract

Designing an antibody with the desired affinity to the antigen is challenging, often achieved by lengthening the hydrophobic CDRs, which can lead to aggregation and cause major hindrance to the development of successful biopharmaceutical products. Aggregation can cause immunogenicity, viscosity and stability issues affecting both the safety and quality of the product. As the hydrophobic residues on the CDR are required for direct binding to antigens, it is not always possible to substitute these residues for aggregation-reduction purposes. Therefore, discovery of specific excipients to prevent aggregation is highly desirable for formulation development. Here, we used a combination of in silico screening methods to identify aggregation-prone regions on an aggregation-prone therapeutic antibody. The most aggregation-prone region on the antibody was selected to conduct virtual screening of compounds that can bind to such regions and act as an aggregation breaker. The most promising excipient candidate was further studied alongside plain buffer formulations and formulations with trehalose using coarse-grained molecular dynamics (CGMD) simulations with MARTINI force field. Mean interaction value between two antibody molecules in each formulation was calculated based on 1024 replicates of 512 ns of such CGMD simulations. Corresponding formulations with an excipient:antibody ratio of 1:5 were compared experimentally by measuring the diffusion interaction parameter *k_D_* and accelerated stability studies. Although the compound with the highest affinity score did not show any additional protective effects compared with trehalose, this study proved using a combination of in silico tools can aid excipient design and formulation development.

## 1. Introduction

Monoclonal antibodies (mAbs) have emerged as a major class of therapeutic agents. Their continued success in clinical and translational science and research has led to discovery of highly potent antibodies are capable of treating a wide range of conditions. Antibody therapies are usually administered via injectable routes such as intravenous (IV), subcutaneous (SC) and intramuscular (IM) route. Many therapeutic mAbs were originally developed for the IV administration route, but IV administration is inconvenient for both patients and healthcare professionals. IV formulations usually involve patient-adjusted dosage calculations and aseptic preparation of infusion volumes. IV administration is also an invasive procedure that requires a specific trained personnel giving the treatment conducted in dedicated infusion facilities, and monitoring during and after treatment can lead to additional hospitalisation and costs to the healthcare system [1]. A single-use fixed-dosed injection device that can be reliably and safely used by patients is more favourable because it reduces hospitalisation, permitting flexibility in dose administrations and making appointments with clinicians [2]. 

In view of providing a treatment that is less invasive and easier to administrate for patients, mAb therapies and reliable devices utilising SC and IM routes for drug delivery are developed. In particular, the SC route is of growing interest for the administration of mAbs. However, the small interstitial spaces for fluids limits the injection volumes, usually 1–2 mL with SC administration [3]. Similarly, IM injections deliver molecules below the subcutaneous space and typically injection volumes for IM injections are limited to 5 mL [4]. For most mAb therapeutics, a relatively large dose (i.e., 8 mg/kg) is often required to achieve therapeutic effects. Therefore, high concentration liquid formulations of mAb solutions suitable for SC/IM delivery are desirable due to the relatively low limit on the injection volume. Certain mAb molecules were found to be highly viscous and exhibit aggregation at concentrations above 100 mg/mL affecting safety, formulation stability and hence shelf-life of the product.

Excipients and stabilisers are commonly added to mAb formulations to modify viscosity, reducing aggregation and to prevent them from degradation. The choice and composition of these excipients and stabilisers are crucial for the performance of the process and the quality of the product. Formulation development is a time and resource consuming process to work out the optimal excipient composition as well as formulation buffer characteristics, such as pH and ionic strength. The selection of excipients also depends on the regulatory requirements. In practice, excipients are commonly selected from existing compendial excipients. As some new mAbs are more challenging to formulate, there is an increasing demand for discovering novel excipients to be more specific and effective preventing aggregation and improving solubility.

Aggregation is considered to be the predominant pathway of degradation, resulting in biological inactivation [5,6], inducing immunogenic responses in patients and result in self-immunity to the therapeutic protein causing loss of therapeutic effects [7]. Aggregation is governed by self-association factors such as hydrogen bonds, hydrophobic interactions, electrostatic and van der Waals forces. Most self-associated mAb species do not affect the folding of the therapeutic protein, and are often reversible through dilutions. However, aggregates can grow into larger stable irreversible species and undergo structural deformation from stable complexes, which can continue to grow into higher order aggregates such as soluble filaments, soluble agglomerated aggregates and macroscopic aggregates [8,9]. Therefore, preventing the self-association of mAb molecules can potentially delay the aggregation process. 

Computational modelling techniques have already been used extensively to assist the development of mAb candidates to help identify problematic sequences based on calculations of their local charges [10] and hydrophobicity [11]. These problematic areas could be engineered to lower their charges and hydrophobicity, reducing viscosity and aggregation propensities. Molecular docking strategies have also been used to support drug discovery where a large number of ligands are docked against a protein target to study their interactions and identify potential drug candidates. Proteins are dynamic in nature and can exist in different conformations. Molecular dynamics (MD) simulations have the advantage of simulating the dynamic processes of structural changes of the protein target when interacting with the ligand. MD simulations can therefore potentially improve the accuracy of prediction.

Veurink and co-workers used the aggregation-prone regions of bevacizumab and found that dexamethasone can prevent formation of bevacizumab dimers at LYS445 located on the Fc region, which interacts with the Fab on the second bevacizumab [12]. Experimentally, dexamethasone was shown to reduce the formation of bevacizumab dimers, trimers and higher order aggregates after 28 days at 40 °C compared with bevacizumab alone. Dexamethasone is a potent corticosteroid, which is associated with a range of side-effects such as masking of infections, blood disorders, adrenal suppression, Cushing’s syndrome, sodium retention with oedema, weight gain, diabetes mellitus, hypertension, hypercholesterolemia and hypertriglyceridemia. Given the increased risk associated with dexamethasone compared with existing excipients, dexamethasone is not an ideal excipient candidate unless there is a clear clinical evidence of co-formulating bevacizumab with dexamethasone. Nerveless, this raises the interesting question of whether it is possible to use computational strategies to screen for specific excipient for a specific antibody that prevents aggregation, preserve product quality and hence reducing the potential failure in development. 

This article presents a computational study which is complemented with experimentation to discover a specific excipient that targets an aggregation-prone region on a specific mAb (mAb1). Three dimensional structural data of the Fv fragment of mAb1 was previously resolved from X-ray diffraction [13] and was used in this study to calculate the most solvent accessible hydrophobic area and commercially available small molecules were screened with molecular docking tool against this area in a view to disrupt mAb-mAb interactions. The molecule with the highest binding affinity was further examined using molecular dynamics simulations to study interactions between mAb1 and the proposed excipient candidate. Formulations of mAb1 with proposed excipient candidate were prepared and compared with standard excipient trehalose. The anti-aggregation effect of the proposed excipient was measured with Dynamics Light Scattering (DLS) and Size-exclusion Chromatography (SEC) after storage at elevated temperature.

## 2. Materials and Methods

mAb1 was obtained from AstraZeneca (Cambridge, United Kingdom), D-(+)-trehalose dihydrate, phosphate buffered saline and glacial acetic acid were purchased from Sigma-Aldrich (Poole, United Kingdom), proposed excipient (Compound **X**) was purchased from Life Chemicals Europe GmbH (Munich, Germany), sodium acetate trihydrate and sodium chloride were purchased from Fisher Chemicals (Loughborough, United Kingdom). 

mAb1 was supplied in 50 mM sodium acetate, 100 mM sodium chloride at pH 5.5. Absolute concentration was determined by measuring the absorbance at 280 nm in triplicate using NanoDrop (ThermoFisher Scientific, Loughborough, UK) calculated with an extinction coefficient of 1.68 mL mg^−1^ cm^−1^ prior to use. Formulations of mAb1 with trehalose and with Compound **X** were prepared by dissolving the corresponding excipient in freshly prepared buffer and added to the mAb1 stock formulation to achieve an Antibody:Excipient molar ratio of 1:5. Subsequently, all samples were prepared with pH adjustment followed by filtering through 0.22 μm sterile filters.

**Identification of aggregation-prone region:** Spatial Aggregation Propensity (SAP) [11] was employed to calculate the hydrophobicity of exposed surface patches of mAb1 from its crystal structure (PDB ID: 5JZ7) [13]. SAP calculations can provide a more detailed analysis of the surface features of the molecule including side-chain hydrophobicity and solvent accessible surface area [11].

(1) $$SAP_{{atom}_{i}}=\sum_{\begin{array}{c} Simulation\;\\ average \end{array}}\left\{\sum_{\begin{array}{c} Residue\;with\;\\ at\;least\;one\;\\ atom\;within\;\\ R\;of\;atom\;i \end{array}}\left[\frac{\begin{array}{c} SAA\;of\;the\;side\;chain\;atoms\;\\ within\;radus\;R\\ \times Residue\;hydrophobicity \end{array}}{\begin{array}{c} SAA\;of\;side\;chain\;atoms\;\\ of\;fully\;exposed\;residue \end{array}}\right]\right\}\;$$

where *SAP* is the Spatial Aggregation Propensity score, *SAA* is the solvent accessible area, *R* is the radius from the central atom and residue hydrophobicity is obtained from the Black and Mould hydrophobicity scale [14].

A short 60 ns MD simulation was prepared for SAP calculations using the X-ray crystallography structure of mAb1 and performed on GROMACS 2016.3 [15] with CHARMM36 topologies [16]. The system was solvated with TIP3 waters and neutralised with 28 NA and 28 CL ion molecules, which is equivalent to 100 mM NaCl. This forms a rectangular box of 7.77 × 7.77 × 7.77 nm³. The overall simulation system has a total 46569 atoms. The system was first minimised and equilibrated for 1 ns. Subsequently, the MD simulation was ran at 2 fs/step with the use of periodic boundary conditions, constant temperature of 300 K and pressure at 1 bar using V-rescale thermostat and Parrinello-Rahman barostat, respectively. Electrostatic and van der Waals interactions were calculated using a Verlet cut-off-scheme with Potential-shift-verlet as modifier with a cut-off of 1.0 nm. Snapshots were taken every 0.01 ns, which are used for SAP calculations. SAP values of mAb1 were calculated at R = 10 Å over a 60 ns MD simulation.

**Molecule selection and virtual screening:** The virtual screening in this study was conducted using AutoDock Vina [17] which is among the most popular and widely used free and open source software for protein–ligand docking. Ideally, the proposed excipient should be soluble in water and have a small molecular weight. Therefore, the following selection criteria have been applied.

1. Currently in stock with a supplier;
2. Log P below 1 to ensure solubility;
3. A molecular weight below 400 g/mol;
4. Chemically unreactive;
5. Overall net charge of 0 at pH 7 was selected to ensure the effect of the ligand as a potential excipient in formulation can bind to the hydrophobic CDR via hydrophobic interactions and van der Waals forces.

This yield a subset of 83845 compounds and input structures in pdbqt format were downloaded from ZINC database [18]. The structures of sucrose, maltose and trehalose were also downloaded from the ZINC database for comparisons. The search grid focused on the most hydrophobic part of mAb1 from the SAP calculation within a cubic box size set at 25 grid points for all three dimensions extended from the Cα atom that has the highest SAP score. The exhaustiveness of the global search was set at 125 to emphasise docking accuracy and number of generated binding modes set to 9. Results were pooled together and ranked.

**CGMD simulations to determine antibody-antibody interactions:** Antibody–antibody interactions were simulated using X-ray crystallography structure of mAb1 Fv fragments. Antibody dimer structure prediction using all-atom representations are prohibitive as they require enormous of computational resources to achieve meaningful timescale and convergence, disassociation rate of mAb molecules may be too low to allow unbinding and binding events to occur [19]. Therefore, CGMD using MARTINI representations was used to reduce the computational resources, allowing for simulation with longer time-scales and representable replicates. 

DAFT protocol [19] was used to aid the conversion of atomistic structure to MARTINI protein force field version 2.2 [20,21] with ElNeDyn [22] applied on the mAb1 Fv fragments using a cut-off distance of 0.9 nm and a force constant of 500 kJ mol^−1^ nm^−2^. In each simulation, two randomly rotated mAb1 Fv fragments were placed at an initial distance of 3 nm and a total of 1024 different relative starting orientations were generate for each set of simulation. A total of three sets of simulations were prepared, two mAb1 Fv fragments, two mAb1 Fv fragments with ten molecules of trehalose and two mAb1 Fv fragments with ten molecules of Compound **X**. All of these simulations were solvated with standard MARTINI water and ionised to 100 mM NaCl using insane [23]. Each simulation systems contained two mAb1 fragments, roughly 9150–9300 standard MARTINI water beads, 68 NA+ ion beads and 66 CL- ion beads [23] in a box of dimensions approximately 13.00 × 13.00 × 6.70 nm³. For systems containing trehalose and Compound **X**, these excipient molecules were added to individual systems by replacing standard MARTINI water beads with GROMACS 2016.3 through a bash script [14]. Topology and force field parameters for trehalose were taken directly from the MARTINI force field parameters for carbohydrate [24]. The topology and force field parameters of Compound **X** were based on atomistic simulations of Compound **X**.

Atomistic topology of Compound **X** was generated with Automated Topology Builder 2.2 (ATB) [25]. Reference atomistic simulation was performed using the GROMOS 54A7 force field [26]. The MD simulation system was prepared and ran using GROMACS 2016.3 [14]. A molecule of Compound **X** was surrounded with 1683 molecules of SPC water in a box of dimensions 3.75 × 3.75 × 3.75 nm³. The system was first minimised and equilibrated for 1 ns and subsequently the production MD simulation was ran at 2 fs/step for a total of 100 ns with a snapshot taken every 1000 steps. Periodic boundary conditions were used along with constant temperature of 300 K and pressure at 1 bar using V-rescale thermostat and Parrinello–Rahman barostat, respectively. Electrostatic and van der Waals interactions were calculated using a Verlet cut-off-scheme with Potential-shift-verlet as modifier with a cut-off of 1.4 nm. 

**Figure 1:**
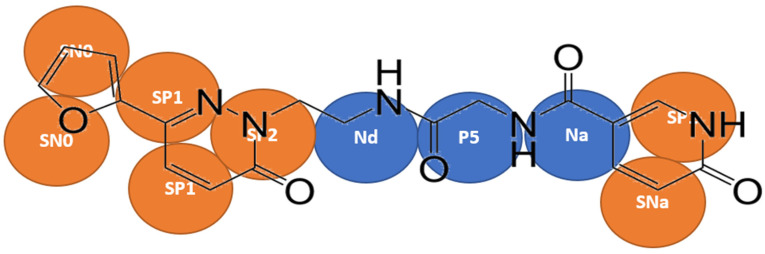
Coarse-grained mapping and bead assignments for Compound **X**. Ordinary MARTINI beads were coloured in blue and S MARTINI beads were coloured in orange.

A two/three-to-one mapping approach was followed for the aromatic rings in Compound **X**, and four-to-one mapping was used for the remainder of the molecule (Figure 1). Bonded interactions were derived by mapping the all-atom simulation to CG resolution, the bond length, angle and dihedral angle between virtual particles over the entire simulation were measure by constructing histograms of respective bond or angle. In most cases, a gaussian distribution with a single peak was obtained. However, in some cases, the distribution may contain more than a single peak, so the most prominent peak was used.

All simulation systems were minimised with martinate [27] using GROMACS 2016.3 [15] for 500 ps at 1 fs/step before undergoing a 100 ps NPT equilibration MD simulation at 20 fs/step. The final production runs were performed with a time step of 20 fs for 512 ns with a snapshot taken every 25,000 steps. Periodic boundary conditions were used, a constant temperature of 300 K was maintained and pressure was controlled at 1 bar using V-rescale thermostat and Berendsen barostat, respectively. Electrostatic and van der Waals interactions were calculated using a Verlet cut-off-scheme with a Potential-shift-verlet as modifier with a cut-off of 1.8 nm. 

**Dynamic Light Scattering:** DLS measurements were performed on a DynaPro PlateReader II system (Wyatt Technology, Santa Barbara, CA, USA) with an 826.1 nm laser using a Corning 3540 384-well non-binding clear flat-bottom polystyrene plate (Corning, New York, NY, USA). Formulations were diluted with filtered fresh buffer for DLS measurements to antibody concentrations of 1, 2, 3, 4, 5, 6, 7, 8, 9 and 10 mg/mL. Samples of all tested formulations in each concentration were added to sextuplicate wells and each well contained 30 µL of the sample. Twenty acquisitions were collected for each well over 5 s. Only particles with hydrodynamic radius between 2 nm and 30 nm were considered for analysis.

(2) $$D=D_{0}\;\left(1+k_{D}c\right)$$

where *k_D_* is the diffusion interaction parameter, c is the concentration of the mAb, *D* is the diffusion coefficient of mAb at concentration *c*, and *D*_0_ is the diffusion coefficient of the antibody molecules in infinite dilution. This equation assumes a linear relationship between diffusion coefficient and concentration of mAb.

**Size-exclusion Chromatography:** Samples for SEC measurements were prepared by first diluting to achieve antibody concentrations of 1 mg/mL and were sealed in glass vials and placed in a 50 °C incubator for 28 days. Samples for measurements were taken immediately after sample preparation, and then after 7 and 28 days of storage. SEC was performed on an Agilent ZORBAX Bio Series GF-250 4 µm column (Agilent Technologies, Santa Clara, CA, USA) on the Agilent Technology 1200 series HPLC system (Agilent Technologies, Santa Clara, CA, USA) at a constant flow rate of 0.5 mL/min with a sample loading of 20 µL and UV detection at 280 nm. The mobile phase consisted of phosphate buffered saline at pH 7.4.

## 3. Results

**Figure 2:**
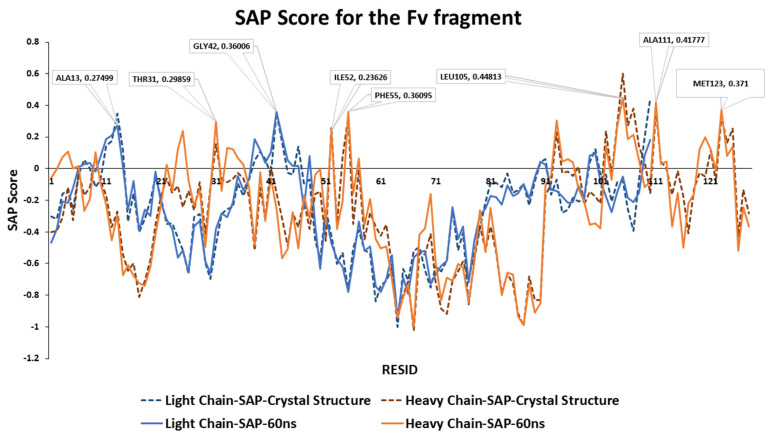
Residue-average SAP scores calculated at R = 10 Å for mAb1 Fv fragment crystal structure and the final snapshot after a 60 ns MD simulation. Residues with SAP scores calculated from the structure at 60 ns greater than 0.15 were highlighted. The SAP scores of individual residues are similar in the crystal structure and the snapshot of the MD simulation at 60 ns. LEU105 on the heavy chain has the highest calculated SAP score within the Fv fragment.

Aggregation-prone regions usually exhibit an enrichment in hydrophobic sequences. In the case of mAb1, at the CDR3 loop (ILE102-LEU109) on the heavy chain contains the largest hydrophobic patch within the Fv fragment with LEU105 showed the highest overall SAP score (Figure 2). Other exposed hydrophobic patches have been identified, including the CDR1 loop (THR31), the CDR2 loop (ILE52-PHE55) and the last β-sheet strand within the Fv fragment (MET123). 

**Figure 3:**
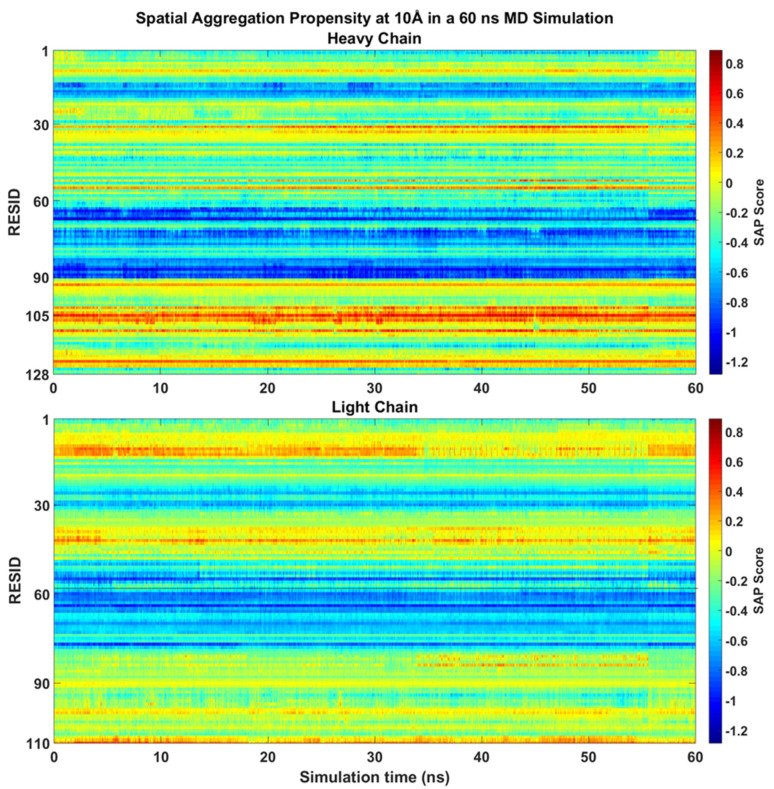
SAP values calculated at R = 10 Å over a 60 ns all-atom MD simulation. The SAP score for most residues within the Fv fragment remained stable the simulation with LEU105 on the heavy chain identified as the most solvent-accessible hydrophobic patch on the Fv fragment.

During the 60 ns all-atom MD simulation, the SAP scores of each residues of mAb1 Fv fragment were similar to the static molecule and no major changes been observed (Figure 3). LEU105 within the CDR3 of the heavy chain remains the residue with the highest SAP score. There were slight increases in SAP scores from the CDR1 loop after 25 ns into the simulation with no significant changes in SAP score in other residues on the heavy chain observed. Only hydrophobic regions surrounding THR43 and ALA13 on the light chain have shown a prominent positive signal, but their intensities were reduced after 40 ns compared to the static crystal structure. 

**Figure 4:**
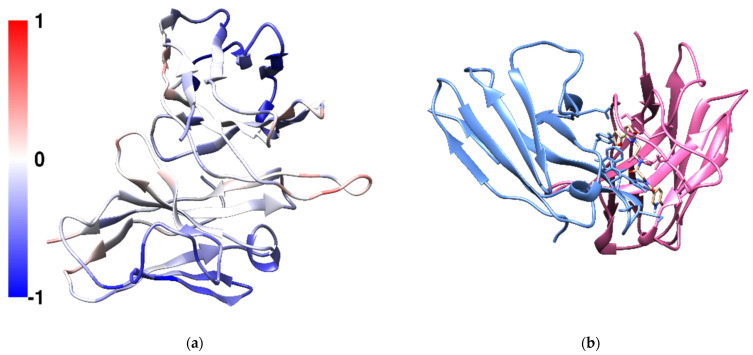
(**a**) Visual representation of the SAP calculation results for mAb1 Fv fragment. The molecule is oriented with the heavy chain shown at the top of the model and the light chain at the bottom with CDRs at the right side. Residues with positive and negative SAP scores were coloured in red and blue, respectively. In general, the light chain is more hydrophilic than the heavy chain with the CDR3 on the heavy chain exhibiting the highest SAP score. (**b**) The configuration of Compound **X** docked to mAb1 Fv fragment with a predicted binding affinity of −8.1 kcal/mol. The heavy chain was coloured in pink and the light chain was coloured in light blue.

The predicted binding affinities of the screened 83846 compounds were between −8.1 and −1.8 kcal/mol. N-{2-[3-(furan-2-yl)-6-oxo-1,6-dihydropyridazin-1-yl]ethyl}-2-[(6-oxo-1,6-dihydropyridin-3-yl)formamido]acetamide (Compound **X**, CPX) was found to have the greatest affinity. Common disaccharides excipients in protein formulations such as trehalose, sucrose and maltose showed weak affinities towards the CDR3 of the heavy chain with trehalose achieving a greater affinity towards the CDR3 region compared with sucrose and maltose (Table 1). Compound **X** was predicted to bind to the CDR3 on heavy chain in a pocket located between the heavy chain and the light chain (Figure 4b). Compound **X** contains three aromatic hydrophobic rings, a furan, a pyridazine and a pyridine. It was predicted to form six hydrogen bonds between the ligand and mAb1 in the docked structure. The carbonyl oxygen on the pyridine ring forms a hydrogen bond with THR110 on the heavy chain and the hydrogen atom of the pyridine amine forms another hydrogen bond with SER97 on the light chain. The carbonyl oxygen on the central glycine forms two hydrogen bonds with ASN31 and ASN32 on the light chain. Within the same amide group, the hydrogen atom on the amide forms a hydrogen bond with ASP104 on the heavy chain. The second nitrogen on pyridazine ring forms a hydrogen bond with TYR112 on the heavy chain. The 4-(furan-2-yl)pyridazine moiety is also π-stacked with TYR103 on the heavy chain.

Compound **X** in MARTINI parameters were developed using the bottom up approach, where the atomistic MD simulation data were mapped into virtual beads and the CG parameters were derived and refined through systematically modifying the individual terms until a high degree of agreement to the atomistic simulation data is achieved. The selection of MARTINI beads were chosen based on the principles of the original MARTINI publication [20]. The focus here is to reproduce the bonded parameters, bond length, angles and dihedral terms. The final parameters of Compound **X** in MARTINI force field file is included in Supplementary Information (S1).

**Figure 5:**
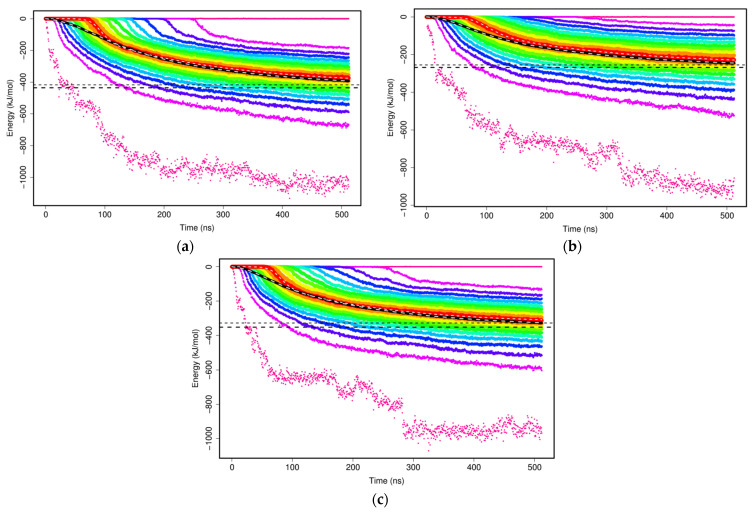
Interaction energy between mAb1 Fv fragment dimers in CGMD simulations. The calculated plateau value of the means for mAb1 Fv fragment dimerisation were determined to be (**a**) −417.2 kJ/mol in 100 mM NaCl, (**b**) −253.1 kJ/mol in 100 mM NaCl with 10 molecules of Compound **X** and (**c**) −319.18 kJ/mol in 100 mM NaCl with 10 molecules of trehalose.

The non-bonded interactions of two mAb1 Fv fragments were evaluated with the sums of Lennard–Jones and Coulomb potentials within the MARTINI CG model to study the formation of mAb1 Fv fragment dimers. Two mAb1 Fv fragments were separated at a defined distance of 3 nm at the beginning of the simulations and there were no interactions at this distance. As the simulations progress, two mAb Fv fragments diffuse and interact with other solutes in the simulations and cause interaction energies between two mAb1 Fv fragments to shift towards negative as binding is thermodynamically favourable (Figure 5). The calculated plateau values of each set of simulations offer a simple overview of how the mAb1 Fv fragment-mAb1 Fv fragment interaction energies interfered by addition of Compound **X** and trehalose (Table 2).

**Figure 6:**
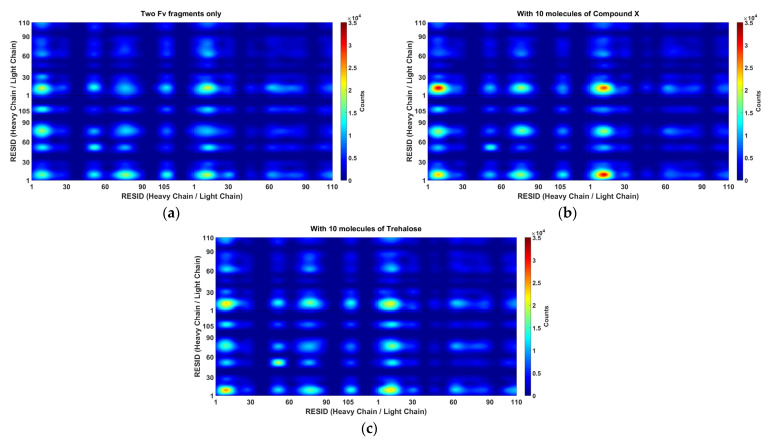
Common binding pairs in a set of 1024 MARTINI CG simulations (**a**) with just two mAb1 Fv fragments in 100 mM NaCl, (**b**) with 10 molecules of Compound **X** and (**c**) with 10 molecules of Trehalose. The occurrence of interaction pairs were coloured according to the colour bar on the right. Main self-association events occur in the CDR1 near residue GLU16 on the light chain. Apart from LEU105, alternative interaction hot-spots were also identified, including LYS13, PHE55 and SER75 on the heavy chain. The occurrences of interaction events the most solvent-accessible hydrophobic patch (LEU105 on heavy chain) slightly reduces with the addition of Compound **X**. Please note that MARTINI representation was based on four-to-one mapping and this graph was generated based on interactions between individual beads not by residue so the number of interaction events may appear differently to Figure 8.

**Figure 7:**
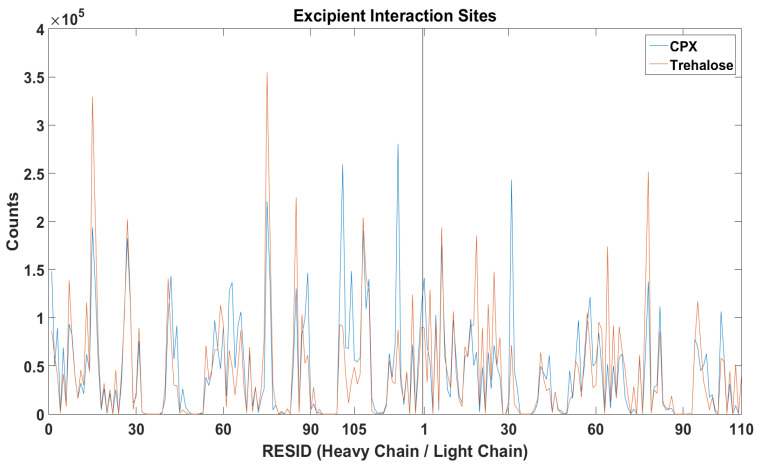
The locations of Compound **X** and trehalose were tracked with respect to the mAb1 Fv fragment Compound **X** was found to bind to LEU105 on heavy chain more frequently than trehalose.

It is important to understand the formation of Fv fragment dimer complexes as interruptions to self-associations via these specific interaction sites will prevent stable dimer complexes formations and hence aggregation. The distance between any pair of beads of different mAb1 Fv fragment molecules was calculated (Figure 6) and the relative locations of excipients were also tracked bases on their centre of masses for all frames for all simulations within a simulation set (Figure 7).

In the simulations without addition of excipients, self-associations through heavy chain LEU105 were not the most favourable. Common self-association events were observed in these MD simulations near residue GLN16 on the light chain and LYS13, PHE55 and SER75 on the heavy chain. From a short 60 ns all-atom simulation (Figure 3), these regions on the heavy chain, especially regions around LYS13 and SER75 have shown much lower SAP scores and were not considered as aggregation-prone regions.

A reduction of dimerisation events through LEU105 on the heavy chain were observed in the presence of Compound **X**, the intended area of interaction as defined in the virtual screening study. The docking position have identified that Compound **X** forms a hydrogen bond with ASP104 and π-stacked with TYR103 on the heavy chain. A small reduction of interaction events through these two residues were also observed. Compound **X** can potentially acts as a mAb-mAb interaction breaker to prevent self-association through the CDR3 of the heavy chain. The addition of Compound **X** caused an increase of interactions between two Fv fragments at GLN16 on the light chain and LYS13 and SER75 on the heavy chain. Interestingly, Compound **X** demonstrated to reduce interactions between two Fv fragments at PHE55 on the heavy chain but not trehalose.

**Figure 8:**
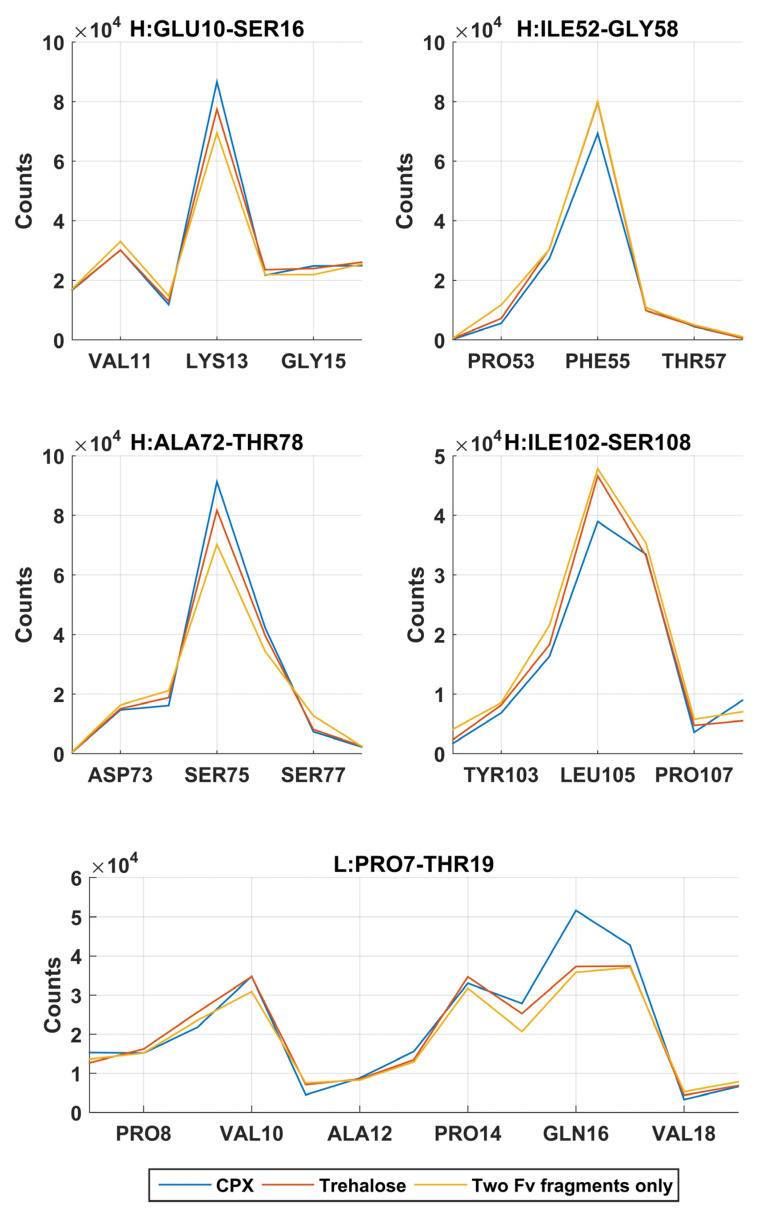
The common interaction locations in a set of 1024 MARTINI CG simulations in under three conditions (1) two mAb1 Fv fragments in 100 mM NaCl, (2) with 10 molecules of Compound **X** and (3) with 10 molecules of Trehalose. Selective excipient Compound **X** binds to the most solvent-accessible hydrophobic patch at LEU105 on the heavy chain and is shown to reduce antibody–antibody interactions via this residue while increasing interactions through GLU16 on the light chain and LYS13 and SER75 on the heavy chain.

In the earlier docking study, trehalose showed a weak affinity to the region close to the CDR3 on heavy chain. In MD simulations with trehalose, no reduction in self-association events through heavy chain LEU105 compared to the simulations with just two Fv fragments. Similar to the case of Compound **X**, the number of interaction events through VAL10 on the light chain and VAL11 on the heavy chain increases with the presence of trehalose. There was also a smaller increase in interaction events through PHE55 on the heavy chain observed. Both Compound **X** and trehalose do not appear to have strong tendencies to bind to these regions so they cannot act as interaction breakers at these sites (Figure 8). Trehalose was found interacting with THR76 on the heavy chain more than any other residues within the Fv fragment. A further decrease in dimer complex formation through the same residue was observed in simulations with trehalose compared to simulation with Compound **X**.

Experimentally, a net attractive force exists between mAb1 molecules in concentrations ranging between 1 and 10 mg/mL as suggested by negative *k_D_* values in all formulations (Table 3). mAb1 was formulated in 50 mM sodium acetate, 100 mM sodium chloride at pH 5.5 and the *k_D_* was determined to be −9.03 mL/g. The addition of Compound X at mAb1:CPX molar ratio of 1:5 resulted in an increase of the *k_D_* value to −6.29 mL/g, suggesting that the intermolecular attractions were reduced compared with the original mAb1 formulation. The reduced mAb-mAb interactions can lead to reduction of viscosity of the solution and likely lower the probability of aggregation.

**Figure 9:**
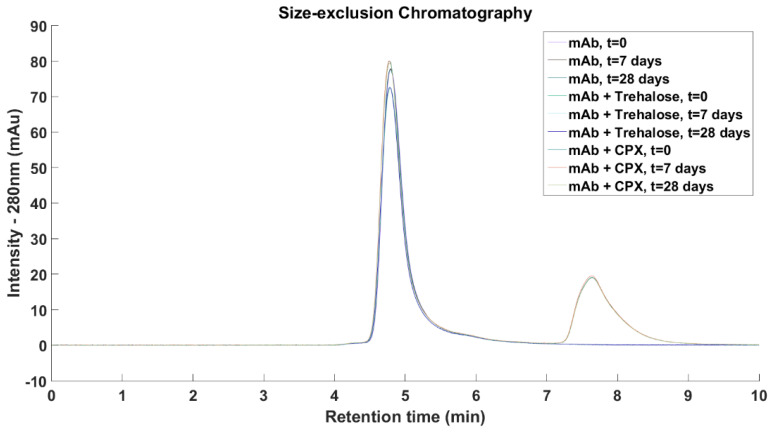
mAb1 and Compound **X** were eluted after 4.8 min and after 7.6 min, respectively. Only a single antibody peak was seen in all tested samples with no higher molecular weight aggregates were detected as well as the intensity and integrity of the mAb1 peaks were maintained over the observed period, suggesting mAb1 stable at 50 °C at 1 mg/mL over 28 days. Neither Compound **X** nor trehalose destabilise mAb1.

mAb1 and Compound **X** were eluted after 4.8 and 7.6 min, respectively. Compound **X** contains three aromatic rings and it showed absorbance at 280 nm while trehalose and other excipients were not detected at this wavelength. No higher molecular weight species were detected across all three formulations and the relative concentration of mAb1 remained constant upon storage at 50 °C for 7 days and 28 days (Figure 9).

## 4. Discussion

In this work, we use the hydrophobicity of the exposed surface patches of the mAb1 Fv fragment to calculate the SAP score to predict the aggregation prone regions. In general, the light chain is less hydrophobic compared with the heavy chain where most of its SAP values were negative. Solvent-accessible hydrophobic loops, especially the CDRs loops are flexible and the hydrophobic residues located within these loops which are responsible for interacting with the target molecule. These structural features can also cause Fab–Fab interaction and pave a pathway to aggregation [11,12,13]. Hydrophobic residues on the CDR loops may not be ideal candidates for mutagenesis because they are responsible for the binding to the target. Therefore, designing an excipient that targets these flexible and the hydrophobic residues remained desirable as they can act as antibody–antibody interactions breakers without modifying the amino acid sequence. In this proof of concept work, it is hoped to examine if antibody–antibody interactions can be reduced with a small molecule that binds preferentially to the most aggregating-prone region on an antibody molecule.

The CDR3 of the heavy chain showed the strongest aggregation propensities within the whole molecule in both static structure and during MD simulation. Aggregation is an unavoidable phenomenon for mAbs and substituting pro-aggregating residues within the CDR can affect biological potency and activity. As an alternative approach, using a specific excipient that prevents Fv fragment–Fv fragment interactions and delaying aggregation while not affecting the binding to the target is highly desirable. Aggregates can induce immunogenic responses, resulting in self-immunity to the therapeutic protein causing loss of therapeutic effects [7]. Hence, it may be justifiable to use a novel excipient like Compound **X** to reduce viscosity and aggregation, improve product characteristics, and most importantly the long-term safety of the mAb.

Disaccharides are hydrophilic in nature and their calculated logPs are smaller than −3 [28], hence they do not exhibit high affinity towards the hydrophobic site via van der Waals interactions. Even Compound **X** gives a greater affinity than disaccharides, it still considered as weak compared to typical affinities of antibody binding to its target [13] so it is reasonable to suggest that binding of Compound **X** to mAb1 will not affect the binding of mAb1 to its target, as it is more energetically more favourable. Although the library selected for screening filtered out compounds with a calculated logP above 1, Compound **X** does not exhibit high aqueous solubility with around 1 mg/mL of the compound solubilised in water at pH 7. Introducing a complex novel excipient like Compound **X** into clinical trial alongside the mAb would require additional safety considerations. Therefore, using Compound **X** in pre-formulating mAb1 needs to be well evaluated by appropriate physicochemical studies and show significant benefit in risk reduction.

Consistent, rapid, accurate and reproducible in silico models are always preferred. However, all-atom MD simulations to probe multi-body interactions requires enormous computational power. Coarse-graining using the MARTINI force field with the aid of DAFT protocol [19] to facilitate generating replicates with different unbiased starting orientations have provided an effective protocol for simulating mAb–mAb interactions in presence of different excipients. The dimerisation of two mAb1 Fv fragments resulted in a wide distribution of interaction energies in each set of simulations. When Compound **X** or trehalose binds to a mAb Fv fragment at the interface of the dimer, these molecules act as physical barriers between two Fv fragments and hence reduce direct interaction surface areas between Fv fragments. Therefore, the addition of excipients of either Compound **X** or trehalose molecules resulted in reduction of interaction energies. Compound **X** was demonstrated to reduce the interaction energies further than trehalose. This may be due to the relative larger size of Compound **X,** which is represented with a 10-bead model, whereas trehalose is represented by a 6-bead model, hence the size of the physical barriers are larger.

It is important to note that surface charges of the antibody molecule also play an important role in mAb-mAb interactions, therefore aggregation does not only depend on hydrophobicity and shape complementarity. Surface charges on the mAb molecule can be masked at high salt concentrations, promoting hydrophobic interactions between protein molecules [29]. SAP calculations focus on identifying solvent exposed hydrophobic patches and effect of surface charges were not considered during the initial binding site selection. Compound **X** was selected in the virtual screening using the structural data of CDR3 loop on the heavy chain as the target. In the MD simulations, Compound **X** was found to bind to the CDR3 on the heavy chain more frequently than trehalose, especially at ARG101 on the heavy chain.

Compound **X** was designed to selectively binding to the most hydrophobic part on the antibody molecule and decreases dimer complex formation through the same residue while increase antibody–antibody interactions through other parts of the antibody molecule in MD simulations. In contrast, trehalose did not show any preference to any of the interaction hot-spots identified and following more closely to the interaction pattern shown with just two Fv fragments alone. Both Compound **X** and trehalose do not appear to show preferential binding towards the LEU105 on the heavy chain. The number of interaction events decreased marginally after the addition of Compound **X**, so it is not considered in the CGMD model to be an effective interaction breaker for LEU105 at the molar ratio of 1:5. This can be due to the simulation time was not long enough to permit Compound **X** to reach the desired binding pocket.

Experimentally, the addition of trehalose at mAb1:trehalose at the same molar ratio resulted in a *k_D_* of −6.33 mL/g, which is very similar to the determined *k_D_* value for the formulation with Compound **X**. Trehalose is commonly added to formulations at a much higher molar ratios to prevent aggregation [30], so a higher concentration may be required to achieve optimum *k_D_* values. Therefore, it is reasonable to suggest that both Compound **X** and trehalose can reduce the attractive interactions between mAb1 molecules at a low antibody:excipient ratio and this low ratio leaves sufficient room for other excipients should the formulation require (i.e., surfactants). However, this DLS study only revealed the strength of global mAb1-mAb1 interactions and did not provide information regarding how Compound **X** or trehalose affects the conformational stability of mAb1 compared to buffer-only conditions, especially the effects of these excipients on aggregation-prone regions previously identified.

This study used a 1 mg/mL for conducting all these stability studies under thermal stress which is also used by Arora and co-workers [31]. The IgG1 mAb used in their work have resulted up to 17% loss in monomer contents formulated with 53 mM of m-Cresol after 28 days storage at 50 °C compared to 1% loss in monomer contents without any addition of antimicrobial preservatives. mAb1 has proven to be a very stable molecule and the addition of Compound **X** or trehalose do not appear to destabilise the molecule. In addition, marketed therapeutic mAb products do not seem to be adversely affected in terms of aggregation by thermal stress at 50 °C. Out of five marketed therapeutic mAbs products, Avastin, Erbitux, Remicade, MabThera and Herceptin, only Erbitux showed significant aggregation after storage at 50 °C for 24 h while the amounts of aggregates in other products were much lower [32]. The degradation kinetics may be dependent on the amino acid sequence, the three-dimensional structure of the molecule and the final compositions of the mAb formulation.

## 5. Conclusions

An ideal excipient should be able to reduce the aggregation-prone interaction between mAb molecules and prevent aggregation to evolve. This study investigated the potential using the structural information of aggregation-prone region of an antibody to search for an excipient aiming to prevent mAb–mAb interactions. Based on the estimated affinities from the docking studies, commonly used disaccharides, such as trehalose, have weak binding affinities compared with the proposed excipient, Compound **X** towards the most hydrophobic area on the tested antibody. Further testing with CGMD has provided an effective protocol for simulating mAb–mAb interactions in the presence of other excipients. This study showed that the dynamic nature of mAb, effects of the charges and realistic formulation environments have to be taken into account when studying mAb–mAb interactions and the exposure of the largest solvent-accessible hydrophobic patch is not the sole consideration for mAb–mAb interactions.

Compound **X** was also tested experimentally and its effect as an anti-aggregation excipient compared with a common excipient, trehalose. A reduction of mAb–mAb interactions in formulations with Compound **X** was seen, but the measured *k_D_* value was similar to formulations with trehalose. mAb1 at 1 mg/mL appears to be very stable with no higher molecular weight aggregates were detected after 28 days storage at 50 °C. Therefore, both Compound **X** and trehalose do not appear to destabilise mAb1. Since the addition of Compound **X** or trehalose have achieved similar experimental results, the use of Compound **X** cannot be justified in mAb formulations as there is currently no safety data to support its use.

Nevertheless, this study described a promising approach for designing tailored excipients to interact preferentially with precisely those local regions on mAb molecule involved in aggregation. Based on the experience gained in this study, the protocol reported here provides support in discovering innovative excipients for mAbs, potentially in conjunction with an automated algorithm for parametrisation of small organic compounds in MARTINI force field [33] for development of a fully automatic CGMD excipient screening process.

## Figures and Tables

**Table 1:** Docking energies of disaccharides and Compound **X** against the CDR3 of the mAb1 heavy chain. Binding of Compound **X** to mAb1 is more energetically favourable towards the calculated aggregation- prone region compared to commonly used disaccharide excipients. Hence, the Compound **X** can act as a selective aggregation breaker on mAb1 CDR3.

Compound	Predicted Best Binding Affinity (kcal/mol)
Trehalose	−5.6
Sucrose	−4.8
Maltose	−4.8
Compound **X**	−8.1

**Table 2:** The calculated plateau value from the mean interaction energy distributions in different systems. Addition of trehalose cause increase in the mean interaction energies and a higher mean interaction energies achieved in systems with Compound **X**, suggesting self-interactions of mAb1 Fv fragments are energetically less favourable in systems with trehalose or Compound **X**.

Excipient Compound Included in MD Simulation	Calculated Mean Interaction Energies (kJ/mol)
-	−417.2
10 Compound **X** molecules	−253.1
10 Trehalose molecules	−319.8

**Table 3:** *k_D_* values of all three formulations of mAb1 were negative suggesting net attractive interactions between antibody molecules in these formulations. Addition of either Compound **X** or trehalose made the *k_D_* values less negative, suggesting self-interactions of mAb1 reduce in prescience of these excipient molecules. Therefore, the propensity for antibody self-interaction decreases with the addition of either Compound **X** or trehalose. Since the *k_D_* values are similar in mAb1 formulation with Compound **X** to those with trehalose, it is reasonable to suggest that Compound **X** is an aggregation breaker non-inferior to trehalose.

Condition	*k_D_D*_0_	*D*_0_ (cm^2^/s)	*k_D_* (mL/g)
mAb1 only	−4.05 × 10^−6^	4.48 × 10^−7^	−9.03
mAb1 with Compound **X**	−2.82 × 10^−6^	4.48 × 10^−7^	−6.29
mAb1 with Trehalose	−2.85 × 10^−6^	4.50 × 10^−7^	−6.33

## Data Availability

All related data and methods are presented in this paper and Supplementary Materials. Additional inquiries should be addressed to the corresponding author.

## Supplementary Materials

The following supporting information can be downloaded at: https://www.mdpi.com/article/10.3390/antib11020040/s1, S1: MARTINI Force Field Parameters for Compound **X**.
