# Supplementary material for: Discovering Novel Small Molecule Compound for Prevention of Monoclonal Antibody Self-Association
Source: Antibodies (Basel). 2022 Jun 8;11(2):40. doi: 10.3390/antib11020040 (PMC9219649; doi:10.3390/antib11020040)
Supplement: Supplementary file 1 [file antibodies-11-00040-s001.zip › antibodies-1732256-supplementary.pdf]

Supplementary Information

MARTINI Force Field Parameters for Compound X

```
[moleculetype]
; molname      nrexcl
  CPX          2

[atoms]
; id   type   resnr  residue  atom   cgnr   charge
   1   P5     1   CPX    P01   1    0
   2   Na     1   CPX    N01   2    0
   3   SP1    1   CPX    S01   3    0
   4   SNa    1   CPX    S02   4    0
   5   Nd     1   CPX    N02   5    0
   6   SP2    1   CPX    S03   6    0
   7   SP1    1   CPX    S04   7    0
   8   SP1    1   CPX    S05   8    0
   9   SN0    1   CPX    S06   9    0
   10  SN0    1   CPX    S07   10   0

[bonds]
; i j    funct    length    force.c.
  1 2      1       0.382     15000
  1 5      1       0.304     15000
  5 6      1       0.280     15000

[constraints]
;  i   j    funct   length
   2   3    1     0.301
   3   4    1     0.125
   2   4    1     0.314
   6   7    1     0.122
   7   8    1     0.210
   6   8    1     0.204
   9  10    1     0.165
   8  9     1     0.245

[angles]
; i j k        funct   angle   force.c.
```

```
 3 2 1     2    180   125.0
 4 2 1     2    120   150.0
 2 1 5     2    95    125.0
 1 5 6     2    150    60.0
 5 6 8     2    130    100.0
 5 6 7     2    160   125.0
 6 8 9     2    150    250.0
 7 8 9     2    180    250.0
 8 9 10    2    180    1000.0

[dihedrals]
; i   j   k   l   funct   angle  force.c.
```
